# Supplementary material for: Identification of Freezing-Responsive microRNAs and Their Targets in Chinese Jujube by Small RNA and Degradome Sequencing
Source: Int J Mol Sci. 2026 Sep 4;27(17):7912. doi: 10.3390/ijms27177912 (PMC13566890; doi:10.3390/ijms27177912)
Supplement: Supplementary file 1 [file ijms-27-07912-s001.zip › Supplementary Material S2/novel_miR_119.pdf]

Provisional ID	:	novel_miR_119
Score total	:	1.8
Score for star read(s)	:	-1.3
Score for read counts	:	0
Score for mfe	:	1.5
Score for randfold	:	1.6
Score for cons. seed	:	
Total read count	:	540
Mature read count	:	540
Loop read count	:	0
Star read count	:	0

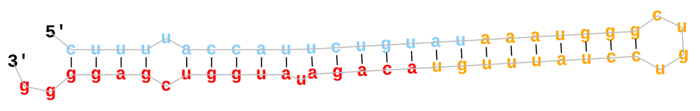

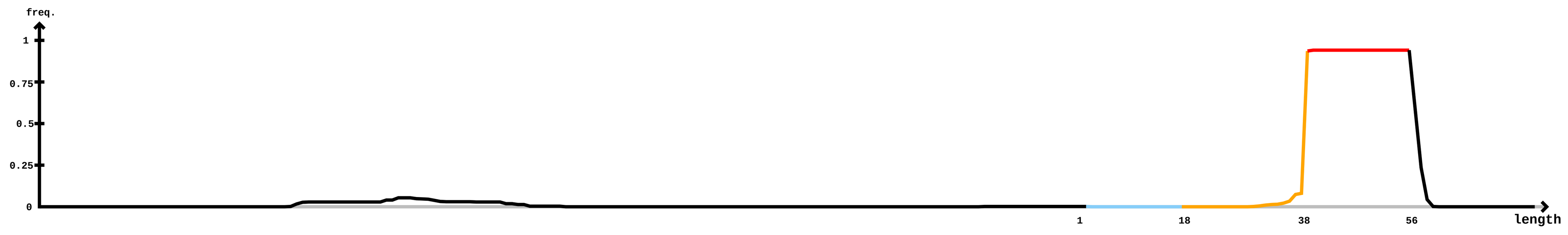

Star Mature

```
5'- uucuugaccagaagaccaagagcuaccuauugagacuucagucuaucuggacacauacuucgugaaaugguuuaugcauguugguuacuugaagcaacaagaucaaguuagaaggauugaacuaucucuccguuuuucuauucauuucuaugauugaugaucauagagggcccuuuuaccauucuguauaaaugggcuguccuauuuguacagauauggucgaggggcacauucaauccuuguuugccu -3'   exp
    ..((((((((((((((...(((((........)).)))..)))).)))))..........((((((....))))))..(((((.((.....)).)))))...)))))....((((((((((((......................((((((((((...)))))))))))((((((.((((((((((((((((((....))))))))))))))).))))).)))))))...))))))))))).......   reads   mm   sample
    ........................................gucuaucCggacacauacuucgugaa.......................................................................................................................................................................................   1   1   JCK-2
    .........................................ucuaucCggacacauacuuc............................................................................................................................................................................................   1   1   JCK-1
    .........................................ucuaucCggacacauacuucg...........................................................................................................................................................................................   1   1   JCK-2
    .........................................ucuaucCggacacauacuucg...........................................................................................................................................................................................   2   1   JCK-3
    .........................................ucuaucCggacacauacuucgu..........................................................................................................................................................................................   1   1   LT-1
    .........................................ucuaucCggacacauacuucguga........................................................................................................................................................................................   4   1   LCK-3
    ..........................................cuaucCggacacauacuuc............................................................................................................................................................................................   2   1   LT-3
    ..........................................cuaucCggacacauacuucgug.........................................................................................................................................................................................   2   1   JCK-2
    ..........................................cuaucCggacacauacuucgug.........................................................................................................................................................................................   2   1   JCK-3
    ...........................................uaucuggacacauacuucgugaaauggu..................................................................................................................................................................................   1   0   LT-2
    ........................................................acuucgAgaaaugguuuaug.............................................................................................................................................................................   1   1   LT-3
    ........................................................acuucgAgaaaugguuuaugcaug.........................................................................................................................................................................   4   1   JT-2
    ........................................................acuucgAgaaaugguuuaugcaug.........................................................................................................................................................................   2   1   JT-3
    ..........................................................uucgugaaaugguuuaug.............................................................................................................................................................................   5   0   LT-2
    ..........................................................uucgugaaaugguuuaugca...........................................................................................................................................................................   3   0   LT-2
    ..............................................................ugaaaugguuuaugcauguuUguu...................................................................................................................................................................   1   1   JT-3
    ..............................................................ugaaaugguuuaugcauguuUguu...................................................................................................................................................................   1   1   JT-2
    ...........................................................................................................................................................gaugaucauagagggccc............................................................................   1   0   JCK-3
    ........................................................................................................................................................................................................ccuauuuguacagauauggucgaggggC.....................   1   1   LT-1
    .........................................................................................................................................................................................................cuauuuguacagauauggucgagggg......................   2   0   LT-1
    ..........................................................................................................................................................................................................uauuuguacagauauggucgagggg......................   3   0   LT-1
    ...........................................................................................................................................................................................................auuuguacagauauggucgagggg......................   2   0   LT-2
    ............................................................................................................................................................................................................uuuguacagauauggucgaggggc.....................   1   0   LT-3
    .............................................................................................................................................................................................................uuguacagauauggucgaggggA.....................   2   1   LT-3
    .............................................................................................................................................................................................................uuguacagauauggucgaggggC.....................   1   1   LT-3
    .............................................................................................................................................................................................................uuguacagauauggucgaggggcaAa..................   1   1   LT-1
    ..............................................................................................................................................................................................................uguacagauauggucgaggggc.....................   1   0   LT-1
    ..............................................................................................................................................................................................................uguacagauauggucgaggggC.....................   5   1   LT-2
    ..............................................................................................................................................................................................................uguacagauauggucgaggggC.....................   1   1   LT-1
    ...............................................................................................................................................................................................................guacagauauggucgagggg......................   1   0   JCK-2
    ...............................................................................................................................................................................................................guacagauauggucgagggg......................   4   0   LCK-3
    ...............................................................................................................................................................................................................guacagauauggucgagggg......................   1   0   LT-3
    ...............................................................................................................................................................................................................guacagauauggucgaggggc.....................   1   0   LT-2
    ...............................................................................................................................................................................................................guacagauauggucgaggggc.....................   1   0   LT-3
```

Star Mature

```
uucuugaccagaagaccaagagcuaccuauugagacuucagucuaucuggacacauacuucgugaaaugguuuaugcauguugguuacuugaagcaacaagaucaaguuaguaaggauugaacuaucucuccguuuuucuauucauuucuaugauugaugaucauagagggcccuuuuaccauucuguauaaaugggcuguccuauuuguacagauauggucgaggggcacauucaauccuuguuugccu
................................................................................................................................................................................................................guacagauauggucgaggggc.....................   1   0   JCK-2
................................................................................................................................................................................................................guacagauauggucgaggggc.....................   3   0   JCK-1
................................................................................................................................................................................................................guacagauauggucgaggggc.....................   2   0   JCK-3
................................................................................................................................................................................................................guacagauauggucgaggggcac...................   2   0   LT-1
................................................................................................................................................................................................................guacagauauggucgaggggcCc...................   1   1   LT-3
................................................................................................................................................................................................................guacagauauggucgaggggcCc...................   7   1   LCK-3
.................................................................................................................................................................................................................uacagauauggucgaggggc.....................   1   0   LT-3
.................................................................................................................................................................................................................uacagauauggucgaggggcC....................   3   1   LT-2
..................................................................................................................................................................................................................acagauauggucgagggg......................   37  0   LCK-3
..................................................................................................................................................................................................................acagauauggucgagggg......................   5   0   JCK-1
..................................................................................................................................................................................................................acagauauggucgagggg......................   30  0   LT-3
..................................................................................................................................................................................................................acagauauggucgagggA......................   4   1   JCK-1
..................................................................................................................................................................................................................acagauauggucgCgggg......................   1   1   LT-1
..................................................................................................................................................................................................................acagauauggucgagggU......................   5   1   LCK-3
..................................................................................................................................................................................................................acagauauggucgagggg......................   19  0   JT-1
..................................................................................................................................................................................................................acagauauggucgagggg......................   30  0   LT-1
..................................................................................................................................................................................................................acagauauggucgagggg......................   2   0   JCK-2
..................................................................................................................................................................................................................acagauauggucgagggg......................   52  0   LT-2
..................................................................................................................................................................................................................acagauauggucgagggA......................   1   1   LT-2
..................................................................................................................................................................................................................acagauauggucgagggg......................   1   0   JCK-3
..................................................................................................................................................................................................................acagauCuggucgagggg......................   1   1   LCK-3
..................................................................................................................................................................................................................acagauauggucgaggggc.....................   4   0   JCK-3
..................................................................................................................................................................................................................acagauauggucgaggggc.....................   19  0   LCK-2
..................................................................................................................................................................................................................acagauauggucgagggCc.....................   2   1   LT-2
..................................................................................................................................................................................................................acagauauggucgaggggA.....................   5   1   LT-3
..................................................................................................................................................................................................................acagauauggucgaggggA.....................   1   1   LT-1
..................................................................................................................................................................................................................acagauauggucgaggggc.....................   19  0   LCK-1
..................................................................................................................................................................................................................acagauauggucgaggggA.....................   4   1   LT-2
..................................................................................................................................................................................................................acagauauggucgagggCc.....................   7   1   LT-1
..................................................................................................................................................................................................................acagauauggucgaggggc.....................   1   0   JCK-1
..................................................................................................................................................................................................................acagauCuggucgaggggc.....................   1   1   LT-1
..................................................................................................................................................................................................................acagauauggucgaggggc.....................   29  0   LT-2
..................................................................................................................................................................................................................acagauauggucgaggggc.....................   7   0   JT-2
..................................................................................................................................................................................................................acagauauggucgagggCc.....................   4   1   LT-3
..................................................................................................................................................................................................................acagauaugUucgaggggc.....................   1   1   LT-1
..................................................................................................................................................................................................................acagauauggucgaggggc.....................   23  0   LT-1
..................................................................................................................................................................................................................acagauauggucgaggggA.....................   6   1   LCK-3
..................................................................................................................................................................................................................acagauauggucgagggCc.....................   13  1   LCK-3
..................................................................................................................................................................................................................acagauauggucgaggggc.....................   32  0   LT-3
..................................................................................................................................................................................................................acagauauggucgaggggc.....................   5   0   JCK-2
..................................................................................................................................................................................................................acagauauggucgaggggc.....................   9   0   LCK-3
..................................................................................................................................................................................................................acagauauggucgaggggc.....................   12  0   JT-3
..................................................................................................................................................................................................................acagauauggucgaggggcC....................   25  1   LT-1
..................................................................................................................................................................................................................acagauauggucgaggggca....................   2   0   LT-3
..................................................................................................................................................................................................................acagauauggucgaggggcU....................   1   1   LT-3
..................................................................................................................................................................................................................acagauauggucgaggggcC....................   14  1   LT-2
..................................................................................................................................................................................................................acagauauggucgaggggcC....................   27  1   LCK-3
..................................................................................................................................................................................................................acagauauggucgagggAca....................   2   1   LT-1
..................................................................................................................................................................................................................acagauauggucgaggggca....................   1   0   LT-1
..................................................................................................................................................................................................................acagauauggucgaggggcC....................   30  1   LT-3
..................................................................................................................................................................................................................acagauauggucgaggggcCc...................   6   1   LCK-3
..................................................................................................................................................................................................................acagauauggucgaggggcCc...................   4   1   LT-2
..................................................................................................................................................................................................................acagauauggucgaggggcCc...................   3   1   LT-1
..................................................................................................................................................................................................................acagauauggucgaggggcCc...................   2   1   LT-3
...................................................................................................................................................................................................................cagauauggucgaggggc.....................   2   0   LT-1
...................................................................................................................................................................................................................cagauauggucgaggggcC....................   1   1   LT-1
```
